# Clinical characteristics and predictors of pulmonary hypertension in chronic obstructive pulmonary disease at different altitudes

**DOI:** 10.1186/s12890-023-02405-8

**Published:** 2023-04-18

**Authors:** Lixia Wang, Faping Wang, Yajun Tuo, Huajing Wan, Fengming Luo

**Affiliations:** 1 Department of Respiratory and Critical Care Medicine, West China Hospital, Sichuan University, Chengdu, Sichuan China; 2 Department of Respiratory and Critical Care Medicine, Qinghai Provincial People’s Hospital, Xining, China; 3 Laboratory of Pulmonary Immunology and Inflammation, Frontiers Science Center for Disease-related Molecular Network, West China Hospital, Sichuan University, Chengdu, Sichuan China

**Keywords:** Chronic obstructive pulmonary disease, Pulmonary arterial hypertension, High altitude, Predictor

## Abstract

**Background:**

Pulmonary hypertension (PH) is a common complication in patients with chronic obstructive pulmonary disease (COPD) and is closely associated with poor prognosis. However, studies on the predictors of PH in COPD patients are limited, especially in populations living at high altitude (HA).

**Objectives:**

To investigate the differences in the clinical characteristics and predictors of patients with COPD/COPD and PH (COPD-PH) from low altitude (LA, 600 m) and HA (2200 m).

**Methods:**

We performed a cross-sectional survey of 228 COPD patients of Han nationality admitted to the respiratory department of Qinghai People’s Hospital (N = 113) and West China Hospital of Sichuan University (N = 115) between March 2019 and June 2021. PH was defined as a pulmonary arterial systolic pressure (PASP) > 36 mmHg measured using transthoracic echocardiography (TTE).

**Results:**

The proportion of PH in COPD patients living at HA was higher than that in patients living at LA (60.2% vs. 31.3%). COPD-PH patients from HA showed significantly different in baseline characteristics, laboratory tests and pulmonary function test. Multivariate logistic regression analysis indicated that the predictors of PH in COPD patients were different between the HA and LA groups.

**Conclusions:**

The COPD patients living at HA had a higher proportion of PH than those living at LA. At LA, increased B-type natriuretic peptide (BNP) and direct bilirubin (DB) were predictors for PH in COPD patients. However, at HA, increased DB was a predictor of PH in COPD patients.

**Supplementary Information:**

The online version contains supplementary material available at 10.1186/s12890-023-02405-8.

## Background

Chronic obstructive pulmonary disease (COPD) is characterized by persistent respiratory symptoms and airflow limitation. It has been listed as one of the top three causes of death worldwide [1]. It is the most common cause of deaths in patients with chronic respiratory disease, with a fatality rate of 0.0419% [2]. Moreover, acute exacerbation of COPD is associated with higher morbidity, mortality and medical costs, aggravating the disease burden [3]. Pulmonary hypertension (PH) is a common complication in patients with COPD, occurring mainly in advanced airflow limitation due to hypoxic vasoconstriction. Additionally, the prevalence of PH in COPD patients depends on the population, the definitions applied, and the tools used to evaluate patients [4, 5]. According to previous studies, the prevalence rate of PH in patients with COPD is approximately 30.0%~70.0% [6]. In addition, PH is associated with an increased risk of exacerbation and mortality in patients with COPD [7, 8]. Climate of HA is characterized by hypobaric hypoxic conditions, coldness, dryness and high ultraviolet light, which causes functional changes in human energy metabolism, neuroendocrine system, hemodynamics and fluid balance, and leading to various diseases [9].

Information about the differences in the predictors of PH in COPD patients between HA and LA was limited, Lei S et al. [10] compared the characteristics of patients with PH between LA and HA, and suggested that patients living at HA had lower BNP and less severe PH than those living at LA. Moreover, Aguirre-Franco C et al. [11] investigated the factors associated with PH in COPD patients living at HA, and showed that GOLD 4 and hypoxemia were the independently associated with PH in COPD patients at HA, however, patients from LA were not enrolled. Furthermore, Lupi-Herrera E et al. [12] suggested that alveolar hypoxia plays a role in producing PH in COPD at HA, and after compared with previous studies, they found that the effect of chronic alveolar hypoxia on PH was attenuated at HA, however, they did not analyze the characteristics of COPD-PH patients and predictors of PH in COPD patients between LA and HA. Therefore, it is reasonable to compare the characteristics and predictors of PH in COPD patients between both regions.

Globally, around 500.3 million people live at HA (defined as ≥ 1500 m above sea-level), [13] and are exposured to hypobaric hypoxia, colder temperatures and drier climates [14]. Some studies have revealed that general populations from HA have greater pulmonary artery systolic pressure (PASP) than those from LA, as measured using transthoracic echocardiography (TTE) [15, 16]. The HA setting combines social-economic factors and environmental conditions which may affect respiratory health. Thus, it is of great significance to recognize the characteristics and predictors of PH in patients with COPD living at HA and LA.

However, information about PH in COPD patients at different altitudes is limited, and some studies have evaluated only the association between PH and COPD, or the prognostic factors of PH in COPD patients [5, 17, 18]. A study conducted in Bogotá (2640 m) have evaluated the prevalence and factors independently associated with PH in COPD patients living at HA, however, only the pulmonary function tests (PFT) and arterial blood gas(ABG) were collected [11]. No study has analyzed the differences in characteristics and predictors for the presence of PH in COPD patients living LA and HA. This study aimed to compare the clinical characteristics and predictors of PH in COPD patients between the Sichuan Plain and the Qinghai Plateau.

## Materials and methods

### Subjects and selection criteria

The cohort was composed of 228 hospitalized patients of Han nationality admitted through the outpatient clinic with a diagnosis of COPD in Qinghai People’s Hospital and West China Hospital of Sichuan University between March 2019 and June 2021, Patients from the two reference centers were selected according to 1:1 pairing by sex and age. Moreover, we collected participants’ demographic characteristics, laboratory tests, PFT and TTE results. The inclusion criteria were all patients with chronic cough or sputum production and the forced expiratory volume in the first second/forced vital capacity ratio (FEV_1_/FVC) post-bronchodilator < 0.7, patients aged ≥ 18 years, and all patients underwent echocardiography. Moreover, we excluded patients with a history of untreated hypertension or other diseases that might affect heart health; PH caused by other diseases (idiopathic PH; connective tissue disease, HIV infection, portal hypertension, congenital heart disease; PH due to left heart disease, silicosis and pulmonary embolism), pregnant and lactating women, and patients with cancer. This study was approved by the Ethics Committee of Qinghai People’s Hospital and West China Hospital of Sichuan University (Ethics number: Review No. 716 of 2021), and conducted following Helsinki’s Declaration. Appropriate consent and assent were obtained from all participants.

### Pulmonary function test (PFT)

Spirometry was performed before and after administering a bronchodilator, and arterial blood gasses (ABG) tests were performed according to the European Respiratory Society (ERS) standardization [19]. Patients were grouped by airflow limitation severity according to the Global Initiative for Chronic Obstructive Lung Disease (GOLD) (mild to moderate: GOLD 1 + 2, severe to very severe: GOLD 3 + 4) [20].

### Transthoracic echocardiography (TTE)

TTE was performed at baseline on admission by an experienced cardiologist using a Philips Sonos 5500® ultrasound machine (Royal Philips, Amsterdam, Netherland). The diagnosis of PH was based on the tricuspid regurgitation peak velocity (TRV). This machine recorded the degree of dilation of right atrium to estimate right atrial pressure (RAP), which was used in the estimation of PASP by the Bernoulli equation: PASP = 4TRV^2^ + RAP (when PASP is > 36 mmHg, PH is considered). Lastly, PH was graded into three groups: mild (36 mmHg < PASP ≤ 45 mmHg), moderate (45 mmHg < PASP ≤ 60 mmHg), and severe (PASP > 60 mmHg) [21–23].

### Statistical analysis

Normally distributed data is represented by a mean ± standard deviation and number (percentage), and skewness distribution data were represented by a median (P_25_, P_75_) and number (percentage). The independent samples t-test and non-parametric test were used to compare measurement data fitting normal and skewed distribution respectively. Additionally, the chi-squared test was used to compare categorical variables. Moreover, univariate and multivariate logistic regression analyses were used to determine the effects of relevant variables on PH in patients with COPD. Skewed distribution variables were transformed into binary variables for logistic regression analysis, and the cut-off value was determined by receiver operating characteristic (ROC) curve analysis. Next, the covariates of multivariate analysis were selected from the univariate analysis with statistical significance (P value < 0.05) and collinearity was excluded (we selected the variables with the strongest correlation with presence of PH to perform logistic regression analysis in the collinear data) (supplement Tables 1 and 2). Lastly, the cut-off value was determined using the ROC curve. SPSS software version 26 (IBM Corporation, Armonk, NY, United States) was used for all the statistical analyses.

## Results

### The baseline characteristics of COPD-PH and COPD-NPH patients from LA and HA

Overall, 115 patients were from LA and 113 from HA. Table 1 lists the baseline characteristics of patients with COPD-PH and COPD without PH (COPD-NPH) living at LA. Patients from LA were mainly men with a mean age of > 65 years. Both groups had similar BMI, somking status and duration of cough (P > 0.05). Another, Table 1 also lists the baseline characteristics of patients with COPD-PH and COPD-NPH patients at HA. Patients from HA were mainly older men. Compared with COPD-NPH patients, COPD-PH patients had lower BMI. Both groups had similar somking condition and duration of cough (P > 0.05).

**Table 1:** Baseline Characteristics of COPD-PH and COPD-NPH patients

Variables	LA			HA		
	COPD-NPH(n = 79)	COPD-PH(n = 36)	P*	COPD-NPH(n = 45)	COPD-PH(n = 68)	P**
male, n(%)	57(72.2)	24(66.7)	0.55	33(73.3)	48(70.6)	0.751
age (years)	66.51 ± 7.89	69.2 ± 8.76	0.098	66.53 ± 10.30	68.53 ± 8.34	0.260
BMI, Kg/m^2^	22.77 ± 3.71	22.64 ± 3.79	0.863	23.35 ± 3.56	21.06 ± 3.91	0.002
overweight/obesity, n(%)	22(27.8)	7(19.4)	0.336	11(24.4)	9(13.2)	0.126
smoking, n(%)	46(58.2)	19(52.8)	0.585	21(46.7)	28(41.2)	0.564
duration of cough, years	10.0(4.0,20.0)	10.0(4.0,20.0)	0.968	5.0(2,7.5)^†^	5.0(3.0,9.5)^§^	0.162
PASP, mmHg	¯¯¯¯	45.00(41.00,61.84)		¯¯¯¯	56.00(47.75,72.75)^§^	
PH stage, n(%)						
mild(36mmHg < PASP ≤ 45mmHg)	¯¯¯¯	18(50.0)		¯¯¯¯	13(19.1)^§^	
moderate to severe(PASP > 45mmHg)	¯¯¯¯	18(50.0)		¯¯¯¯	55(80.9)	

*Comparison among patients with COPD-NPH and COPD-PH at LA

**Comparison among patients with COPD-NPH and COPD-PH at HA.

†Statistical significance was set to 0.05 compared with COPD-NPH patients at LA.

§Statistical significance was set to 0.05 compared with COPD-PH patients at LA.

Abbreviations: LA = low altitude;HA = high altitude;BMI = body mass index;BMI = body mass index; PASP = pulmonary artery systolic pressure

Data are shown as mean ± SD, median (quartile) or n (%).

### The laboratory examination and pulmonary function test of COPD-PH and COPD-NPH patients from LA and HA

We observed significant differences in total bilirubin (TB), indirect bilirubin (IB), BNP, platelet (PLT), and lymphocyte (LY) levels between COPD-PH and COPD-NPH patients living at LA (P < 0.05) (Table 2). COPD-PH patients had higher bilirubin and BNP levels than COPD-NPH patients. Conversely, the PLT and LY levels in COPD-PH patients were lower than those in COPD-NPH patients.

Moreover, we observed significant differences in TB, IB, BNP, serum albumin (ALB), arterial partial pressure of oxygen (PaO_2_) and arterial partial pressure of carbon dioxide (PaCO2), arterial oxygen saturation (SaO_2_), HCO_3_^−^, forced expiratory volume in the first second (FEV_1_), FEV_1_/FVC, and GOLD stages between COPD-PH and COPD-NPH patients from HA (P < 0.05) (Table 2). COPD-PH patients had higher bilirubin, BNP, PaCO_2_, HCO_3_^−^ and GOLD stages than COPD-NPH patients. Conversely, the ALB, PaO_2_, SaCO_2_, FEV_1_, and FEV_1_/FVC levels were lower in COPD-PH patients than in COPD-NPH patients.

**Table 2:** Laboratory examination and pulmonary function test of COPD-PH and COPD-NPH patients

Laboratory examination and pulmonary function test of COPD-PH and COPD-NPH patients						
	LA			HA		
	COPD-NPH(n = 79)	COPD-PH(n = 36)	P*	COPD-NPH(n = 45)	COPD-PH(n = 68)	P**
TB,umol/L	9.55(6.73,12.35)	10.50(8.70,14.20)	0.035	13.10(10.70,16.15)^†^	17.65(11.65,25.05)^§^	0.008
DB, umol/L	2.90(2.20,4.10)	3.80(3.10,4.75)	0.007	2.70(2.10,3.45)	4.55(2.45,6.28)	0.022
IB, umol/L	5.90(4.50,9.23)	6.80(5.50,9.40)	0.049	10.10(8.50,13.25)^†^	13.50(8.75,19.00)^§^	0.021
ALT, IU/L	21.50(12,31.25.00)	15.00(10,27.50)	0.786	18.00(12.00,28.00)^†^	18(13.00,24.50)	0.200
AST, IU/L	20.00(15.705,25)	19.00(15.50,26.00)	0.403	20.00(17.00,27.00)	22.00(19.00,29.00)	0.651
TP, g/L	65.55(60.35,68.45)	63.80(62.45,66.85)	0.638	65.20(61.65,68.25)	63.80(58.28,68.83)	0.087
ALB,g/L	39.67 ± 3.87	39.16 ± 3.36	0.496	39.07 ± 3.63	36.66 ± 3.77^§^	0.001
Cr, umol/L	74.00(60.75,87.00)	71.00(57.50,87.50)	0.838	71.00(62.50,84.50)	71.00(65.00,89.00)	0.769
BNP, pg/mL	76.50(46.50,143.75)	211.00(125.50,1085.00)	< 0.001	37.00(21.50,118.00)^†^	234.50(53.50,396.50)^§^	< 0.001
CRP, mg/mL	5.12(2.61,12.50)	8.92(3.31,19.85)	0.100	2.56(0.67,6.90)^†^	3.47(1.82,6.51)§	0.054
PCT, ng/mL	0.040(0.020,0.073)	0.030(0.020,0.050)	0.755	0.037(0.026,0.049)	0.041(0.026,0.050)	0.786
Hb, g/L	136.67 ± 19.07	137.89 ± 23.60	0.769	167.13 ± 27.27^†^	172.41 ± 30.94^§^	0.354
PLT, x10^9/L	192.10 ± 73.12	163.11 ± 62.47	0.042	165.97 ± 59.83^†^	146.84 ± 51.81	0.074
WBC, x10^12/L	7.16(5.74,8.77)	6.75(5.94,7.69)	0.247	5.54(4.59,7.07)^†^	5.18(4.50,6.51)^§^	0.511
NEUT, x10^9/L	4.46(3.30,6.22)	4.49(3.63,5.51)	0.995	4.79(3.45,5.59)^†^	3.56(2.77,4.47)^§^	0.932
LYx10^9/L	1.61 ± 0.72	1.35 ± 0.52	0.029	1.41 ± 0.47	1.23 ± 0.52	0.064
PH	7.41 ± 0.03	7.42 ± 0.05	0.196	7.42 ± 0.04	7.42 ± 0.04	0.44
PaO_2_, mmHg	80.80(67.33,98.03)	80.70(68.90,120.85)	0.821	77.00(59.00,98.50)^†^	63.50(54.75,77.25)^§^	0.011
PaCO_2_, mmHg	43.95 ± 7.76	42.41 ± 10.34	0.393	39.82 ± 6.69^†^	44.91 ± 8.66	0.002
SaO_2_, %	97.20(94.93,98.43)	96.20(94.60,99.20)	0.767	96.00(91.00,98.00)^†^	92.00(88.00,95.25)^§^	0.013
HCO_3_^−^, mmol/L	25.95(24.18,30.83)	26.70(23.80,29.05)	0.671	25.20(22.85,27.20)	28.50(25.78,30.20)^§^	0.001
FEV_1_, % predicted	46.45(33.10,58.73)	47.50(31.60,66.35)	0.27	57.40(37.40,79.35)	40.50(26.63,50.75)^§^	0.008
FEV_1_/FVC, % predicted	45.63(38.45,60.33)	48.22(40.72,63.20)	0.278	58.94(52.48,63.66)^†^	55.18(44,35,61.23)	0.044
VC, % predicted	73.86 ± 18.61	73.63 ± 19.69	0.952	71.95 ± 23.14	62.98 ± 24.49	0.054
GOLD stages (FEV_1_, % predicted), n%			0.08			< 0.001
GOLD1 + 2(≥ 50% predicted)	28(35.40)	19(52.80)		27(60.00)^†^	18(26.50)^§^	
GOLD3 + 4(< 50% predicted)	51(64.60)	17(47.20)		18(40.00)	50(73.50)	

*Comparison among patients with COPD-NPH and COPD-PH at LA.

**Comparison among patients with COPD-NPH and COPD-PH at HA.

†Statistical significance was set to 0.05 compared with COPD-NPH patients at LA.

§Statistical significance was set to 0.05 compared with COPD-PH patients at LA.

Abbreviations: LA = low altitude;HA = high altitude;TB = total bilirubin; DB = direct bilirubin; IB = indirect bilirubin; ALT = alanine transaminase; AST = glutamic oxalacetic transaminase; Cr = creatinine; TP = total protein; ALB = serum albumin; BNP = brain natriuretic peptide; CRP = C-reaction protein; PCT = procalcitonin; Hb = hemoglobin; PLT = platelet; WBC = white blood cell; NEUT = neutrophil; LY = lymphocyte; pH = potential of hydrogen; PaO_2_ = arterial partial pressure of oxygen; PaCO_2_ = arterial partial pressure of carbon dioxide; SaO_2_ = arterial oxygen saturation; BE = base excess; HCO_3_^−^= concentration of bicarbonate radical; FEV_1_ = forced expiratory volume in the first second; FEV_1_/FVC = forced expiratory volume in the first second/forced vital capacity; VC = vital capacity. GOLD = Global Initiative for Chronic Obstructive Pulmonary Disease

Data are shown as mean ± SD, median (quartile) or n (%).

### The proportion and baseline characteristics of COPD-PH patients from LA and HA

**Fig. 1:**
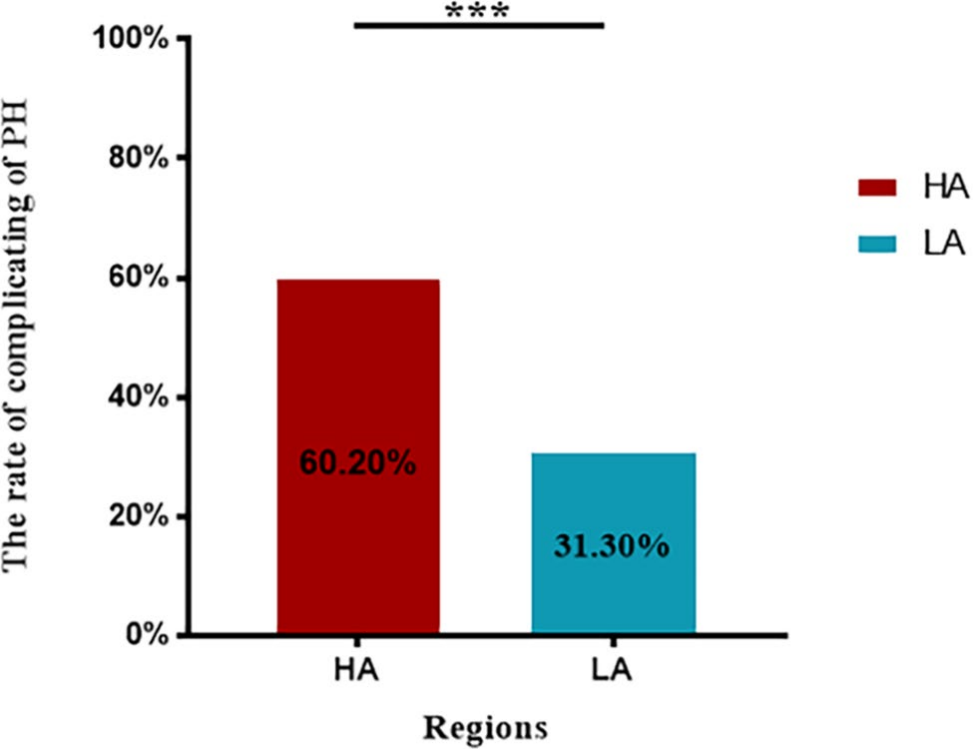
The rate of complicating PH in COPD patients between LA and HA

**Fig. 2:**
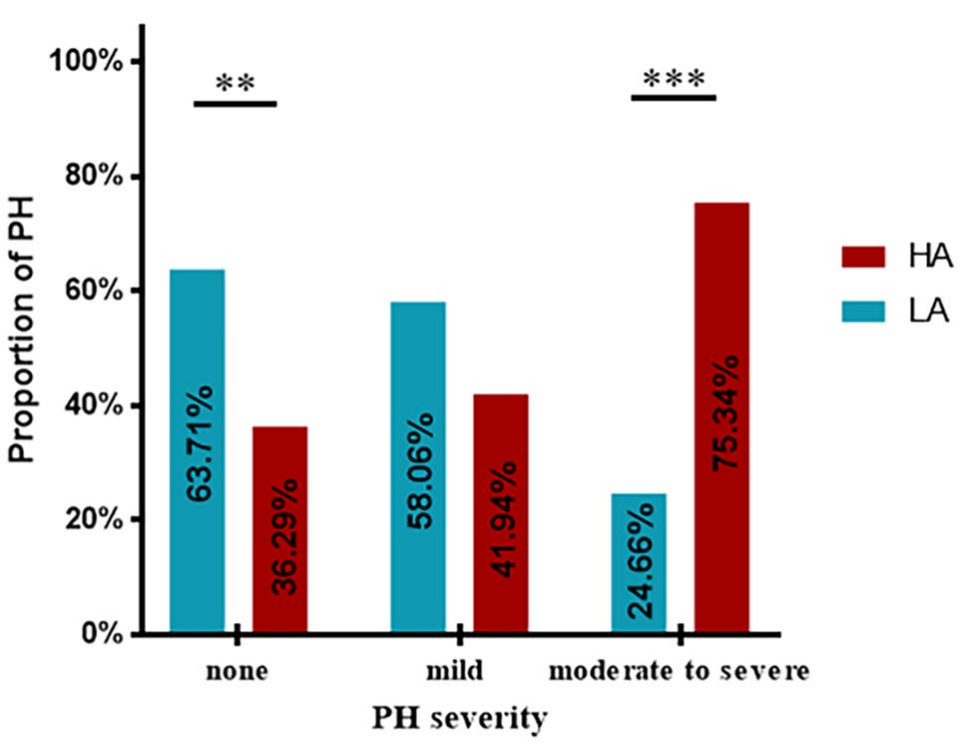
The proportion of PH severity between LA and HA

We enrolled 228 patients from LA and HA. There were 31.3% (36/115) and 60.2% (68/113) of COPD-PH patients from LA and HA, respectively, had PASP > 36 mmHg measured by echocardiography, and the difference was statistically significant (P < 0.001) (Fig. 1). Table 1 presents the baseline characteristics of COPD-PH patients in both groups. As illustrated, both groups of COPD-PH patients were mainly older men with similar BMI (P > 0.05). COPD-PH patients from LA had a longer duration of cough (10.00 years vs. 5.00 years, P = 0.007) and lower PASP (45.00 mmHg vs. 56.00 mmHg, P = 0.003) than those from HA. COPD-PH patients with a mild stage of PH were primarily from LA (58.06%), and those with moderate to severe PH were mainly from HA (75.3%) (P = 0.001). (Fig. 2)

### Laboratory examination and pulmonary function test of COPD-PH from LA and HA

Significant differences were observed in hemoglobin (Hb) levels, white blood cell (WBC) counts, ALB, TB, IB, BNP, C-reaction protein (CRP) levels, FEV_1_, vital capacity (VC), PaO_2_, PaCO_2_ levels and proportion of GOLD 1 + 2 between COPD-PH patients living at LA and HA (P < 0.05). The parameters in COPD-PH patients living at LA, such as WBC counts, ALB, BNP, CRP levels, FEV_1_, VC, PaO_2_, and PaCO_2_ levels, and the proportion of GOLD 1 + 2 were higher than those in patients living at HA. Conversely, Hb, TB and IB levels were lower in COPD-PH patients living at LA than in those patients living at HA. Lastly, we observed no significant differences in PLT and LY counts, DB, alanine transaminase (ALT), glutamic oxalacetic transaminase (AST), total protein (TP), creatinine (Cr), and procalcitonin (PCT) levels, potential of hydrogen (pH), PaCO_2_, base excess (BE) levels, and FEV_1_/FVC levels in COPD-PH patients living in both regions (Table 2).

### Predictors of PH in COPD patients

**Fig. 3:**
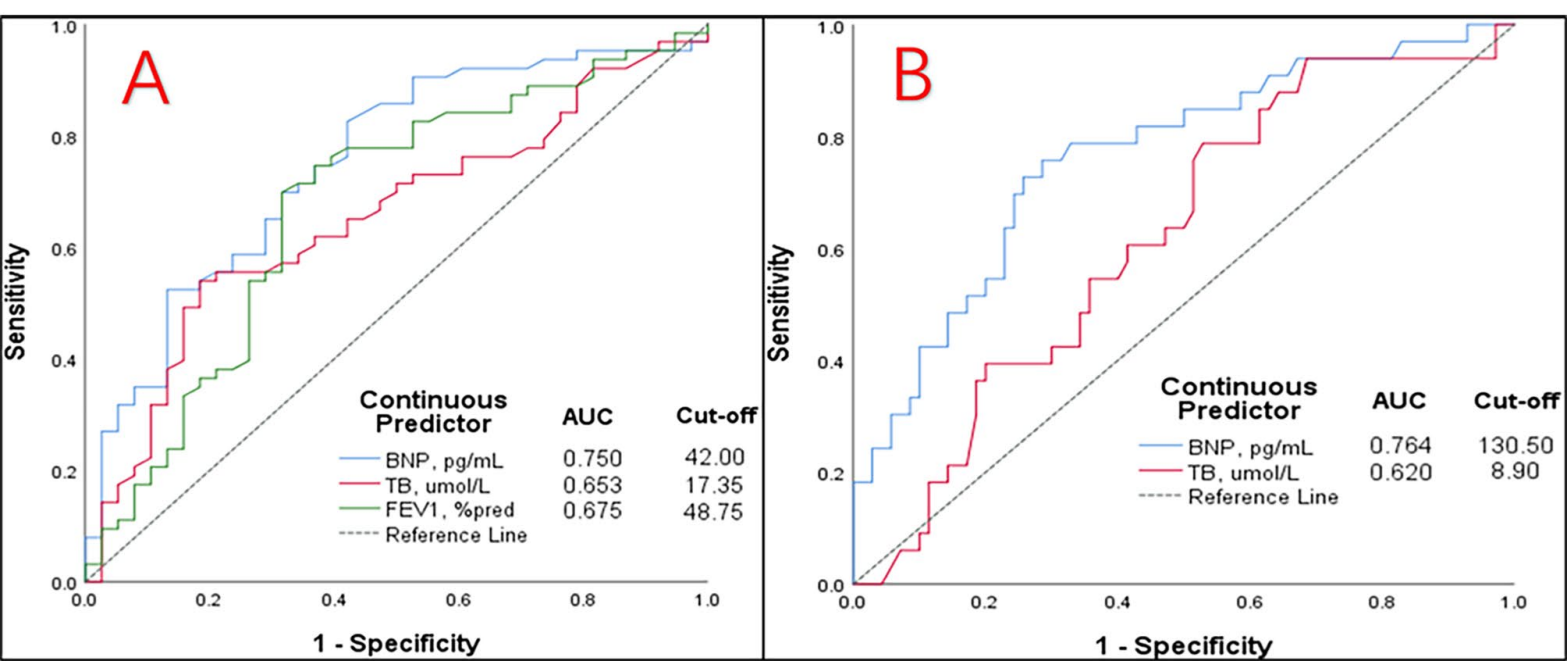
ROC curve of skewed distribution variables for logistic regression analysis and cut-off of the variables. (A) at LA. (B) at HA. ROC, Receiver operator characteristic. AUC, area under the curve

The cut-off values of the skewed distribution variables that for logistic regression analysis were determined using ROC curve analysis (Fig. 3). Moreover, BNP levels in both groups had a maximum area under the curve (AUC). At LA, 130.50 pg/mL was determined as the cut-off for BNP (AUC = 0.764, sensitivity: 75.80%, specificity: 71.40%) in ROC analyses. At HA, 42.00 pg/mL was determined as the cut-off for BNP (AUC = 0.750, sensitivity: 82.50%, specificity: 57.90%), and 48.75%predicted was determined as the cut-off for FEV_1_ (AUC = 0.675, sensitivity: 64.40%, specificity: 70.60%) in the ROC analysis. Information about the other variables of both groups is listed in Table 3.

**Table 3:** Information on ROC curves for continuous Predictor of PH in COPD patients

Information on ROC curves for continuous Predictor of PH in COPD patients						
	LA			HA		
	TB,umol/L	BNP, pg/mL		TB,umol/L	BNP, pg/mL	FEV_1_, %pred
Cut-off	8.90	130.50		17.35	42.00	48.75
Sensitivity(Sens),%	78.80	75.80		54.00	82.50	64.40
Specificity(Spec),%	47.10	71.40		81.60	57.90	70.60
AUC	0.620	0.764		0.653	0.750	0.675

Abbreviations: LA = low altitude;HA = high altitude;TB = total bilirubin;BNP = brain natriuretic peptide;FEV1 = forced expiratory volume in the first second

We evaluated the factors associated with PH in COPD patients using univariate and multivariate logistic regression analyses at HA and LA (Tables 4 and 5). BNP ≥ 130.50 pg/mL (OR: 10) and TB ≥ 8.90 umol (OR:3.3) were predictors of PH in COPD patients from LA after adjusting for PLT (Table 4). TB ≥ 17.35umol (OR:3.4, 95%CI: 1.07–11.14, p = 0.039) was a predictor of PH in COPD patients from HA after adjusting for BNP, ALB, PaCO_2_ and FEV_1_(Table 5). Therefore, increased BNP was a predictor of PH in COPD patients from LA rather than HA, increased TB was a predictor of PH in COPD patients from HA and LA.

**Table 4:** Predictors of PH in COPD patients from LA - Univariable and multivariate logistic regression analysis

Predictors of PH in COPD patients from LA - Univariable and multivariate logistic regression analysis				
Variables	Univariable OR(95%CI)	P	Multivariable OR(95%CI)	P
TB ≥ 8.9, umol/L	3.0(1.2,7.4)	0.017	3.3(1.1,10)	**0.035**
BNP ≥ 130.5, pg/mL	7.8(3.0,20)	< 0.001	10(3.5,28)	**< 0.001**
PLT, x10^9/L	0.99(0.99,1.0)	0.046	0.34(0.104,1.1)	0.07
LY, x10^9/L	0.53(0.28,1.0)	0.054	¯¯¯¯	¯¯¯¯

Abbreviations:TB = total bilirubin; BNP = brain natriuretic peptide; PLT = platelet; LY = lymphocyte

**Table 5:** Predictors of PH in COPD patients from HA - Univariable and multivariate logistic regression analysis

Predictors of PH in COPD patients from HA - Univariable and multivariate logistic regression analysis				
	Univariable OR(95%CI)	P	Multivariable OR(95%CI)	P
BNP ≥ 42.00, pg/mL	6.5(2.6,16)	< 0.001	2.9(0.92,9.4)	0.069
BMI, Kg/m^2^	0.85(0.76,0.95)	0.004	0.88(0.77,1.0)	0.063
TB ≥ 17.35, umol/L	3.7(1.6,8.7)	0.002	3.4(1.1,11)	**0.04**
ALB, g/L	0.84(0.75,0.94)	0.002	0.89(0.77,1.0)	0.136
PaO_2_ < 60, mmHg	1.7(0.76,4.0)	0.195	¯¯¯¯	¯¯¯¯
PaCO_2_, mmHg	1.1(1.0,1.2)	0.003	1.1(0.99,1.2)	0.09
FEV_1_ ≥ 48.75, %pred	0.23(0.10,0.76)	< 0.001	0.41(0.13,1.3)	0.123

Abbreviations:BMI = body Mass Index; TB = total bilirubin; BNP = brain natriuretic peptide; ALB = serum albumin; PaO2 = arterial partial pressure of oxygen; PaCO2 = arterial partial pressure of carbon dioxide; FEV1 = forced expiratory volume in the first second;

## Discussion

Our study showed that patients with COPD living at HA had a higher proportion of PH than those living at LA. Moreover, COPD-PH patients from HA showed significantly higher TB, BNP, Hb, and PASP and a higher proportion of moderate to severe PH. Additionally, they show WBC, PaO_2_, lower duration of cough and poor lung function compared with patients from LA. Multivariate logistic regression analysis indicated that the predictors of PH in COPD patients are different between HA and LA (increased TB vs. increased TB and BNP).

Previous studies showed that the rate of PH in COPD patients and the PASP of COPD-PH patients from HA were higher than those in patients from LA,[11, 15, 24–26] and pulmonary artery pressure was negatively correlated with FEV_1_, [27, 28] consistent with our results. These findings suggested that PH could be induced by HA, [29, 30] is possibly associated with chronic alveolar hypoxia, and is involved in the development of PH in patients with COPD who resided at HA permanently [12]. Moreover, Thabut G et al. [31] showed that FEV_1_ was related to mean pulmonary arterial pressure (mPAP) using univariate analysis consistent with our result. However, Scharf SM et al. [28] found that mPAP was significantly related to FEV_1_ using multivariate analysis. This may be because low PaO_2_ was an exclusion criterion in the study by Scharf SM et al. Moreover, in the studies of Scharf SM et al. [28] and Thabut G et al. [31], mPAP was determined from right heart catheterization in some patients, our PASP data determined from TTE. These results showed that there could be a negative correlation between the prevalence of PH and FEV_1_.

PH was more common in COPD patients with severe (GOLD 3) to very severe (GOLD 4) airflow limitation living at HA, our result (HA: 73.53%, LA:47.22%) was consistent with previous studies, Aguirre-Franco C et al. [11] showed that the proportion of PH in COPD patients with severe to very severe airflow limitation living at an average altitude of 2640 m was 64.00%. However, Jatav V.S et al. [32] reported that the proportion of PH in COPD patients with severe to very severe airflow restriction living at an average altitude of 598 m was 47.30%, suggesting a higher risk of complicating PH for COPD patients living at HA compared with those had similar airflow limitation living at LA. Furthermore, COPD patients with severe airflow limitation are more likely to have PH, possibly because increased airflow limitation could cause more severe hypoxia in COPD patients, resulting in severe hypoxic pulmonary vasoconstriction—the main mechanism of PH [33–35]. Moreover, severe hypoxia leads to endothelial cell injury and impaired vascular regeneration and remodeling, and causing significant proliferation and resistance to pulmonary artery resident cells apoptosis [36–38].

The distinctive feature of our study was that our subjects were all the permanent residents of HA (2200 m) and LA (600 m). Consistent with previous reports, we found that people living at HA had lower PaO_2_, SaO_2_, FEV_1_, VC and higher Hb than those living at LA, [15, 25, 39, 40] probably because of the chronic hypoxic environment of HA. Regarding some inflammation indexes, we found that patients from HA had lower WBC and CRP levels than those from LA. Previous studies reported that general population and patients with thromboembolic disease living at HA had lower WBC than those living at LA [41–43]. One hypothesis is that hypoxia causes increased erythroid activity and relatively decreased myeloid/monocytic lineage activity. Additionally, HA might change plasma volume [41]. Furthermore, CRP was closely associated with WBC levels, and some studies have shown that prolonged hypoxia may resolve inflammation, suggesting the adaptation of vascular endothelium to hypoxia [44]. Regarding the role of inflammation in the development of PH in COPD patients, previous studies have shown that CRP, interleukin (IL)-1, and IL-6 levels were higher in COPD-PH patients than in COPD-NPH patients [45–47]. Grimminger J et al. [48] showed that: (1) chronic inflammation could lead to endothelial dysfunction, reducing the levels of endothelium-derived relaxing factors (for example, nitric oxide and prostacyclin) and increasing the levels of endothelium-derived contracting factors (for example, reactive oxygen species and endothelin). (2) chronic inflammation could lead to loss of ciliary cell function and instability of the airways, leading to COPD and hypoxia, which is associated with endothelial dysfunction. (3) CD8 + T-cell infiltration of the adventitia of the pulmonary arteries could lead to endothelium-dependent relaxation and increased intima thickness. Some studies have suggested that higher inflammation is associated with a worse prognosis in patients with PH, [49, 50] and anti-inflammatory treatments are a promising mitigation strategy for PH [51, 52]. However, no studies have reported differences in the role of inflammation in COPD-PH patients living at HA and LA.

Additionally, we found that the predictors of PH in COPD patients were different between LA and HA. Firstly, the BNP level was higher in COPD-PH patients than in COPD-NPH at both HA and LA; however, increased BNP was the only predictor of PH in COPD patients from LA. BNP is a biomarker secreted by the ventricular muscle that can be used to evaluate cardiac function and prognosis in heart failure and other cardiovascular diseases, and hypoxia can stimulate its release [53, 54]. Therefore, vascular adaptations to hypoxia may determine higher BNP in populations living at HA than those living at LA, consistent with our results (Table 2). However, a previous study on the relationship between PH and altitude arrived at inverse conclusions, it reported that PH patients from LA had higher BNP levels than those from HA [55]. These differences may be because the population had interstitial lung disease, which was different from our subjects; meanwhile, all the subjects were Italians in this previous study. Increased BNP level was associated with higher PASP and mortality in patients with PH, and plasma BNP level could be regarded as a protocol for the early identification of PH [56–58]. Regarding patients living at HA, due to long-term exposure to hypobaric hypoxic environment, their body undergoes some adaptive changes such as hypoxic pulmonary vasoconstriction and ventricular hypertrophy [34, 59]. Therefore, the increased BNP level was not a predictor of PH in COPD patients living at HA.

Furthermore, we found that increased BMI was related to PH in COPD patients living at HA using univariate. Being overweight and obese have a positive association with cardiovascular and all-cause mortality [60]. However, many studies suggested that high BMI was associated with low mortality in COPD patients—the “obesity paradox” [61, 62]. The obesity paradox means that obesity in older patients or in patients with several chronic diseases might be protective and associated with decreased mortality [63]. Moreover, a previous study from Southeast Iran plateau suggested that low BMI was independently associated with severe PH in COPD patients, indicating that high BMI might be a protective factor in patients with severe PH in COPD patients, consistent with our results for patients living at HA [64]. However, our result regarding PH was different from some previous studies, which reported that high BMI was independently associated with PH and BMI was positively correlated with PASP [65, 66]. In our study, BMI was not independently associated with PH in COPD patients from HA. The differences may be because the subjects of the previous studies were the general population and patients without specific background diseases, which were more than 3000 and 8000 subjects, respectively. Moreover, the race in our study differed from that of the previous studies. Additionally, our patients were dwellers at HA, excluding interference from other altitudes.

Moreover, we found that bilirubin levels were higher in COPD-PH patients than in COPD-NPH patients living at HA and LA. Additionally, increased TB was an independent predictor of PH in COPD patients living at HA and LA; COPD-PH patients from HA had significantly higher TB than those from LA. Similarly, some studies on TB and PH reported that PASP levels were positively correlated with TB, and TB was an independent predictor of PH, [67, 68] the reasons could be that HA is characterized by hypoxia and bilirubin is an endogenous antioxidant molecule related to oxidative stress [69, 70]. Moreover, in our study, transaminases between COPD-PH at HA and COPD-PH at LA were not significantly different, indicating that serum bilirubin was more sensitive to hemodynamic changes than transaminases, [71] however, the sensitivity of serum bilirubin and transaminases to altitude changes were required for assessment in more studies.

The highlight of this study was that the subjects were from two reference centers and we reasonably excluded the those with other diseases associated with PH. However, the study has some limitations. Primarily, it was a cross-sectional retrospective study, thus, we could not directly determine causality from the results. Secondly, although we had two reference centers, each altitude had a single reference center and the sample size was limited, therefore, the results could not be reliably extrapolated to the general population with COPD living at similar altitudes. Thirdly, there was no linear correlation between PaO_2_ and PASP at both HA and LA in our study which was different from Scharf SM et al. [28] and Thabut G et al. [31], the main reason could be the small sample size of PH patiens in our study. Fourthly, other diseases with coexisting pathologic conditions that could cause PH were excluded in our study, therefore, we could not classify PH into different subgroups in this study. Moreover, we could not obtain the rate of complicating PH in patients with COPD in different GOLD groups using ABE assessment tool, [72] because the study subjects were hospitalized for COPD exacerbation, and they were all in group E according to GOLD 2023. A previous study had reported a higher rate of complicating PH in group E, [73] however, in our study, we could not compare the difference in the rate of complicating PH between different COPD patients in group A-B and E. Lastly, TTE was used to estimate PASP instead of the right cardiac catheter(RHC) in this study. RHC is superior to TTE in monitoring complete hemodynamic assessment, [74] however, it is invasive, expensive and difficult to use on a large scale [75, 76].

## Conclusion

This study showed that the proportion of PH complications and moderate to severe PH in COPD patients living at HA was higher than that in patients living at LA. Moreover, increased TB was the common predictor of PH in COPD patients living at LA and HA, and increased BNP was only the predictor of PH in COPD patients living at LA. Therefore, multicenter studies and large sample size are needed to explore the differences in predictors of PH in COPD patients living at different altitudes.

## Electronic supplementary material

Below is the link to the electronic supplementary material.

Supplementary Material 1

Supplementary Material 2

## Data Availability

All data generated or analyzed during this study are included in the article and additional file.

## List of Abbreviations

COPD: chronic obstructive pulmonary disease
PH: Pulmonary hypertension
LA: low altitude
HA: high altitude
PASP: pulmonary artery systolic pressure
mPAP: mean pulmonary arterial pressure
TTE: transthoracic echocardiography
PFT: pulmonary function tests
ABG: arterial blood gas
TB: total bilirubin
DB: direct bilirubin
IB: indirect bilirubin
ALT: alanine transaminase
AST: glutamic oxalacetic transaminase
Cr: creatinine
TP: total protein
ALB: serum albumin
BNP: brain natriuretic peptide
CRP: C-reaction protein
PCT: procalcitonin
Hb: hemoglobin
PLT: platelet
WBC: white blood cell
NEUT: neutrophil
LY: lymphocyte
pH: potential of hydrogen
PaO_2_: arterial partial pressure of oxygen
PaCO_2_: arterial partial pressure of carbon dioxide
SaO_2_: arterial oxygen saturation
BE: base excess
HCO_3_^−^: concentration of bicarbonate radical
FEV_1_: forced expiratory volume in the first second
FEV_1_/FVC: forced expiratory volume in the first second/forced vital capacity
VC: vital capacity
GOLD: Global Initiative for Chronic Obstructive Pulmonary Disease
PASP: pulmonary artery systolic pressure
IL: interleukin
RHC: right cardiac catheter

## References

[1] Halpin DMG, Celli BR, Criner GJ, Frith P, López Varela MV, Salvi S (2019). The GOLD Summit on chronic obstructive pulmonary disease in low- and middle-income countries. Int J Tuberc Lung Dis.

[2] Prevalence and attributable health burden of chronic respiratory diseases (2020). 1990–2017: a systematic analysis for the global burden of Disease Study 2017. Lancet Respir Med.

[3] Hurst JR, Vestbo J, Anzueto A, Locantore N, Müllerova H, Tal-Singer R (2010). Susceptibility to exacerbation in chronic obstructive pulmonary disease. N Engl J Med.

[4] McLaughlin VV, Archer SL, Badesch DB, Barst RJ, Farber HW, Lindner JR (2009). ACCF/AHA 2009 expert consensus document on pulmonary hypertension: a report of the American College of Cardiology Foundation Task Force on Expert Consensus documents and the American Heart Association: developed in collaboration with the American College of chest Physicians, american thoracic society, Inc., and the Pulmonary Hypertension Association. Circulation.

[5] Chaouat A, Naeije R, Weitzenblum E (2008). Pulmonary hypertension in COPD. Eur Respir J.

[6] Minai OA, Chaouat A, Adnot S (2010). Pulmonary hypertension in COPD: epidemiology, significance, and management: pulmonary vascular disease: the global perspective. Chest.

[7] McGhan R, Radcliff T, Fish R, Sutherland ER, Welsh C, Make B (2007). Predictors of rehospitalization and death after a severe exacerbation of COPD. Chest.

[8] Blanco I, Tura-Ceide O, Peinado VI, Barberà JA (2020). Updated Perspectives on Pulmonary Hypertension in COPD. Int J Chron Obstruct Pulmon Dis.

[9] Burtscher M (2014). Effects of living at higher altitudes on mortality: a narrative review. Aging Dis.

[10] Lei S, Sun Z, He X, Li C, Zhang Y, Luo X (2019). Clinical characteristics of pulmonary hypertension patients living in plain and high-altitude regions. Clin Respir J.

[11] Aguirre-Franco C, Torres-Duque CA, Salazar G, Casas A, Jaramillo C, Gonzalez-Garcia M. Prevalence of pulmonary hypertension in COPD patients living at high altitude.Pulmonology. 2022.doi:10.1016/j.pulmoe.2021.12.006 10.1016/j.pulmoe.2021.12.006 35151623

[12] Lupi-Herrera E, Sandoval J, Seoane M, Bialostozky D (1982). Behavior of the pulmonary circulation in chronic obstructive pulmonary disease. Pathogenesis of pulmonary arterial hypertension at an attitude of 2,240 meters. Am Rev Respir Dis.

[13] Tremblay JC, Ainslie PN. Global and country-level estimates of human population at high altitude. Proc Natl Acad Sci U S A. 2021;118(18). 10.1073/pnas.2102463118. 10.1073/pnas.2102463118 PMC8106311 33903258

[14] Moore LG (2017). Measuring high-altitude adaptation. J Appl Physiol (1985).

[15] Soria R, Egger M, Scherrer U, Bender N, Rimoldi SF (2016). Pulmonary artery pressure and arterial oxygen saturation in people living at high or low altitude: systematic review and meta-analysis. J Appl Physiol (1985).

[16] Mirrakhimov AE, Strohl KP (2016). High-altitude pulmonary hypertension: an update on Disease Pathogenesis and Management. Open Cardiovasc Med J.

[17] Vizza CD, Hoeper MM, Huscher D, Pittrow D, Benjamin N, Olsson KM (2021). Pulmonary hypertension in patients with COPD: results from the comparative, prospective Registry of newly initiated therapies for pulmonary hypertension (COMPERA). Chest.

[18] Castaldi PJ, Hersh CP, Reilly JJ, Silverman EK (2009). Genetic associations with hypoxemia and pulmonary arterial pressure in COPD. Chest.

[19] Miller MR, Hankinson J, Brusasco V, Burgos F, Casaburi R, Coates A (2005). Standardisation of spirometry. Eur Respir J.

[20] Agustí A, Celli BR, Criner GJ, Halpin D, Anzueto A, Barnes P (2023). Global Initiative for Chronic Obstructive Lung Disease 2023 Report: GOLD Executive Summary. Eur Respir J.

[21] Galiè N, Torbicki A, Barst R, Dartevelle P, Haworth S, Higenbottam T (2004). Guidelines on diagnosis and treatment of pulmonary arterial hypertension. The Task Force on diagnosis and treatment of pulmonary arterial hypertension of the European Society of Cardiology. Eur Heart J.

[22] Chemla D, Castelain V, Humbert M, Hébert JL, Simonneau G, Lecarpentier Y (2004). New formula for predicting mean pulmonary artery pressure using systolic pulmonary artery pressure. Chest.

[23] Simonneau G, Montani D, Celermajer DS, Denton CP, Gatzoulis MA, Krowka M, et al. Haemodynamic definitions and updated clinical classification of pulmonary hypertension. Eur Respir J. 2019;53(1). 10.1183/13993003.01913-2018. 10.1183/13993003.01913-2018 PMC6351336 30545968

[24] Lichtblau M, Saxer S, Furian M, Mayer L, Bader PR, Scheiwiller PM, et al. Cardiac function and pulmonary hypertension in central asian highlanders at 3250 m. Eur Respir J. 2020;56(2). 10.1183/13993003.02474-2019. 10.1183/13993003.02474-2019 32430419

[25] Güvenç TS, Erer HB, Kul S, Perinçek G, Ilhan S, Sayar N (2013). Right ventricular morphology and function in chronic obstructive pulmonary disease patients living at high altitude. Heart Lung Circ.

[26] Dubroff J, Melendres L, Lin Y, Beene DR, Ketai L (2020). High geographic prevalence of pulmonary artery hypertension: associations with ethnicity, drug use, and altitude. Pulm Circ.

[27] Oswald-Mammosser M, Apprill M, Bachez P, Ehrhart M, Weitzenblum E (1991). Pulmonary hemodynamics in chronic obstructive pulmonary disease of the emphysematous type. Respiration.

[28] Scharf SM, Iqbal M, Keller C, Criner G, Lee S, Fessler HE (2002). Hemodynamic characterization of patients with severe emphysema. Am J Respir Crit Care Med.

[29] Sime F, Banchero N, Penaloza D, Gamboa R, Cruz J, Marticorena E (1963). Pulmonary hypertension in children born and living at high altitudes. Am J Cardiol.

[30] Vogel JH, McNamara DG, Hallman G, Rosenberg H, Jamieson G, McCrady JD (1967). Effects of mild chronic hypoxia on the pulmonary circulation in calves with reactive pulmonary hypertension. Circ Res.

[31] Thabut G, Dauriat G, Stern JB, Logeart D, Lévy A, Marrash-Chahla R (2005). Pulmonary hemodynamics in advanced COPD candidates for lung volume reduction surgery or lung transplantation. Chest.

[32] Jatav VS, Meena SR, Jelia S, Jain P, Ajmera D, Agarwal V, et al. Echocardiographic findings in chronic obstructive pulmonary disease and correlation of right ventricular dysfunction with disease severity. Int J Adv Med. 2017;4(2). 10.18203/2349-3933.ijam20171045.

[33] Fayngersh V, Drakopanagiotakis F, Dennis McCool F, Klinger JR (2011). Pulmonary hypertension in a stable community-based COPD population. Lung.

[34] Siques P, Brito J, Pena E (2018). Reactive oxygen species and pulmonary vasculature during hypobaric hypoxia. Front Physiol.

[35] Sydykov A, Mamazhakypov A, Maripov A, Kosanovic D, Weissmann N, Ghofrani HA, et al. Pulmonary hypertension in Acute and Chronic High Altitude Maladaptation Disorders. Int J Environ Res Public Health. 2021;18(4). 10.3390/ijerph18041692. 10.3390/ijerph18041692 PMC7916528 33578749

[36] Bourgeois A, Omura J, Habbout K, Bonnet S, Boucherat O (2018). Pulmonary arterial hypertension: new pathophysiological insights and emerging therapeutic targets. Int J Biochem Cell Biol.

[37] Ranchoux B, Harvey LD, Ayon RJ, Babicheva A, Bonnet S, Chan SY et al. Endothelial dysfunction in pulmonary arterial hypertension: an evolving landscape (2017 Grover Conference Series). Pulm Circ. 2018;8(1):2045893217752912.doi:10.1177/2045893217752912 10.1177/2045893217752912 PMC5798691 29283043

[38] Yuan JX, Rubin LJ (2005). Pathogenesis of pulmonary arterial hypertension: the need for multiple hits. Circulation.

[39] Cross TJ, Wheatley C, Stewart GM, Coffman K, Carlson A, Stepanek J (2018). The influence of thoracic gas compression and airflow density dependence on the assessment of pulmonary function at high altitude. Physiol Rep.

[40] Basak N, Norboo T, Mustak MS, Thangaraj K (2021). Heterogeneity in hematological parameters of high and low Altitude Tibetan populations. J Blood Med.

[41] Alkhaldy HY, Z AA, Abouzaid AA, Elbahaie HM, Al Amoudi SM, Andarawi M (2020). The prevalence of isolated Neutropenia at High Altitude in Southern Saudi Arabia: does Altitude affect leucocyte count?. Int J Gen Med.

[42] Algahtani FH, AlQahtany FS, Al-Shehri A, Abdelgader AM (2020). Features and incidence of thromboembolic disease: a comparative study between high and low altitude dwellers in Saudi Arabia. Saudi J Biol Sci.

[43] Siqués P, Brito J, León-Velarde F, Barrios L, De La Cruz JJ, López V (2007). Hematological and lipid profile changes in sea-level natives after exposure to 3550-m altitude for 8 months. High Alt Med Biol.

[44] Wood JG, Johnson JS, Mattioli LF, Gonzalez NC (1999). Systemic hypoxia promotes leukocyte-endothelial adherence via reactive oxidant generation. J Appl Physiol (1985).

[45] Kwon YS, Chi SY, Shin HJ, Kim EY, Yoon BK, Ban HJ (2010). Plasma C-reactive protein and endothelin-1 level in patients with chronic obstructive pulmonary disease and pulmonary hypertension. J Korean Med Sci.

[46] Eddahibi S, Chaouat A, Tu L, Chouaid C, Weitzenblum E, Housset B (2006). Interleukin-6 gene polymorphism confers susceptibility to pulmonary hypertension in chronic obstructive pulmonary disease. Proc Am Thorac Soc.

[47] Humbert M, Monti G, Brenot F, Sitbon O, Portier A, Grangeot-Keros L (1995). Increased interleukin-1 and interleukin-6 serum concentrations in severe primary pulmonary hypertension. Am J Respir Crit Care Med.

[48] Grimminger J, Ghofrani HA, Weissmann N, Klose H, Grimminger F (2016). COPD-associated pulmonary hypertension: clinical implications and current methods for treatment. Expert Rev Respir Med.

[49] Cerik IB, Dindas F, Koyun E, Dereli S, Sahin A, Turgut OO (2022). New prognostic markers in pulmonary arterial hypertension: CRP to albumin ratio and uric acid. Clin Biochem.

[50] Mazurek JA, Horne BD, Saeed W, Sardar MR, Zolty R (2017). Galectin-3 levels are elevated and predictive of Mortality in Pulmonary Hypertension. Heart Lung Circ.

[51] Rabinovitch M, Guignabert C, Humbert M, Nicolls MR (2014). Inflammation and immunity in the pathogenesis of pulmonary arterial hypertension. Circ Res.

[52] El Alam S, Pena E, Aguilera D, Siques P, Brito J. Inflammation in Pulmonary Hypertension and Edema Induced by Hypobaric Hypoxia exposure. Int J Mol Sci. 2022;23(20). 10.3390/ijms232012656. 10.3390/ijms232012656 PMC9604159 36293512

[53] Casals G, Ros J, Sionis A, Davidson MM, Morales-Ruiz M, Jiménez W (2009). Hypoxia induces B-type natriuretic peptide release in cell lines derived from human cardiomyocytes. Am J Physiol Heart Circ Physiol.

[54] Woods DR, Mellor A, Begley J, Stacey M, O’Hara J, Hawkins A (2013). Brain natriuretic peptide and NT-proBNP levels reflect pulmonary artery systolic pressure in trekkers at high altitude. Physiol Res.

[55] Ruocco G, Cekorja B, Rottoli P, Refini RM, Pellegrini M, Di Tommaso C (2015). Role of BNP and echo measurement for pulmonary hypertension recognition in patients with interstitial lung disease: an algorithm application model. Respir Med.

[56] Goetze JP, Videbaek R, Boesgaard S, Aldershvile J, Rehfeld JF, Carlsen J (2004). Pro-brain natriuretic peptide as marker of cardiovascular or pulmonary causes of dyspnea in patients with terminal parenchymal lung disease. J Heart Lung Transplant.

[57] Leuchte HH, Neurohr C, Baumgartner R, Holzapfel M, Giehrl W, Vogeser M (2004). Brain natriuretic peptide and exercise capacity in lung fibrosis and pulmonary hypertension. Am J Respir Crit Care Med.

[58] Leuchte HH, Holzapfel M, Baumgartner RA, Neurohr C, Vogeser M, Behr J (2005). Characterization of brain natriuretic peptide in long-term follow-up of pulmonary arterial hypertension. Chest.

[59] Pena E, Brito J, El Alam S, Siques P. Oxidative stress, kinase activity and inflammatory implications in right ventricular hypertrophy and heart failure under hypobaric hypoxia. Int J Mol Sci. 2020;21(17). 10.3390/ijms21176421. 10.3390/ijms21176421 PMC7503689 32899304

[60] Lavie CJ, Milani RV, Ventura HO (2009). Obesity and cardiovascular disease: risk factor, paradox, and impact of weight loss. J Am Coll Cardiol.

[61] Spelta F, Fratta Pasini AM, Cazzoletti L, Ferrari M (2018). Body weight and mortality in COPD: focus on the obesity paradox. Eat Weight Disord.

[62] Zafrir B, Adir Y, Shehadeh W, Shteinberg M, Salman N, Amir O (2013). The association between obesity, mortality and filling pressures in pulmonary hypertension patients; the “obesity paradox. Respir Med.

[63] Donini LM, Pinto A, Giusti AM, Lenzi A, Poggiogalle E (2020). Obesity or BMI Paradox? Beneath the tip of the Iceberg. Front Nutr.

[64] Samareh Fekri M, Torabi M, Azizi Shoul S, Mirzaee M (2018). Prevalence and predictors associated with severe pulmonary hypertension in COPD. Am J Emerg Med.

[65] Frank RC, Min J, Abdelghany M, Paniagua S, Bhattacharya R, Bhambhani V (2020). Obesity is Associated with Pulmonary Hypertension and modifies outcomes. J Am Heart Assoc.

[66] Moreira EM, Gall H, Leening MJ, Lahousse L, Loth DW, Krijthe BP (2015). Prevalence of Pulmonary Hypertension in the General Population: the Rotterdam Study. PLoS ONE.

[67] Song X, Yang K, Chen G, Duan W, Yao D, Li S (2021). Characteristics and risk factors of pulmonary hypertension in patients with hyperthyroidism. Endocr Pract.

[68] Xu XQ, Lv ZC, Liu QQ, Zhao QH, Wu Y, Sun K (2017). Direct bilirubin: a new risk factor of adverse outcome in idiopathic pulmonary arterial hypertension. Int J Cardiol.

[69] Arancibia A, Nella Gai M, Paulos C, Chávez J, Pinilla E, Angel N (2004). Effects of high altitude exposure on the pharmacokinetics of furosemide in healthy volunteers. Int J Clin Pharmacol Ther.

[70] Seppen J, Bosma P (2012). Bilirubin, the gold within. Circulation.

[71] Allen LA, Felker GM, Pocock S, McMurray JJ, Pfeffer MA, Swedberg K (2009). Liver function abnormalities and outcome in patients with chronic heart failure: data from the Candesartan in Heart failure: Assessment of Reduction in Mortality and Morbidity (CHARM) program. Eur J Heart Fail.

[72] Global Initiative for. Chronic Obstructive Lung Disease.

[73] Gupta KK, Roy B, Chaudhary SC, Mishra A, Patel ML, Singh J (2018). Prevalence of pulmonary artery hypertension in patients of chronic obstructive pulmonary disease and its correlation with stages of chronic obstructive pulmonary disease, exercising capacity, and quality of life. J Family Med Prim Care.

[74] Forfia PR, Trow TK (2013). Diagnosis of pulmonary arterial hypertension. Clin Chest Med.

[75] Habib G, Torbicki A (2010). The role of echocardiography in the diagnosis and management of patients with pulmonary hypertension. Eur Respir Rev.

[76] Bossone E, Citro R, Ruggiero A, Kuersten B, Gregorio G, Blasi F (2007). [Echocardiography and pulmonary arterial hypertension]. Monaldi Arch Chest Dis.

