# Correction: Clinical characteristics and predictors of pulmonary hypertension in chronic obstructive pulmonary disease at different altitudes

**DOI:** 10.1186/s12890-023-02493-6

**Published:** 2023-06-05

**Authors:** Lixia Wang, Faping Wang, Yajun Tuo, Huajing Wan, Fengming Luo

**Affiliations:** 1 Department of Respiratory and Critical Care Medicine, West China Hospital, Sichuan University, Chengdu, Sichuan China; 2 Department of Respiratory and Critical Care Medicine, Qinghai Provincial People’s Hospital, Xining, China; 3 Laboratory of Pulmonary Immunology and Inflammation, Frontiers Science Center for Disease-Related Molecular Network, West China Hospital, Sichuan University, Chengdu, Sichuan China

**Correction: BMC Pulm Med 23, 127 (2023)**

https://doi.org/10.1186/s12890-023-02405-8

Following publication of the original article [1], it was brought to the publisher's attention that Fig. 3 had been replaced by a duplication of the data of Fig. 2. Figure 3 has now been corrected in the published article and a copy of the figure may be seen in this erratum.

**Fig. 3:**
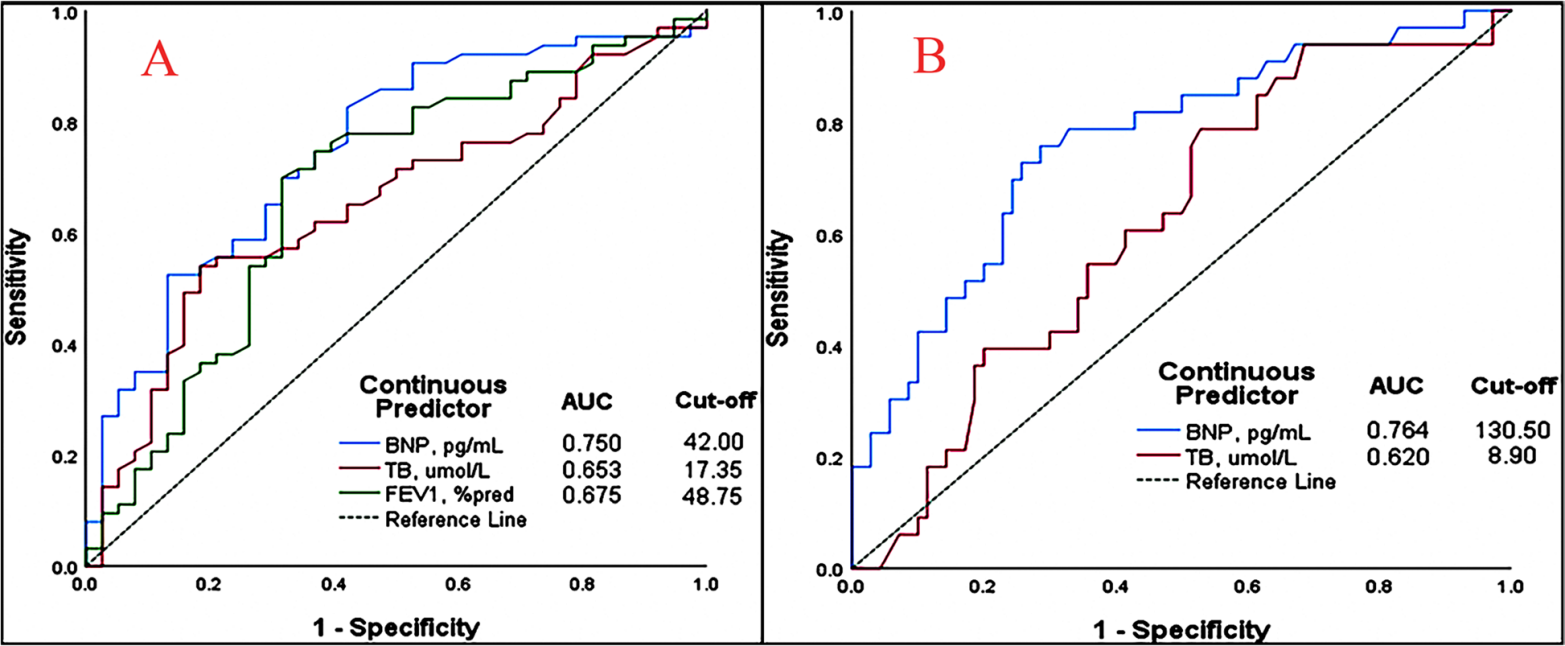
ROC curve of skewed distribution variables for logistic regression analysis and cut-off of the variables. **A** at LA. **B** at HA. ROC, Receiver operator characteristic. AUC, area under the curve

The publisher thanks you for reading this erratum and apologizes for any inconvenience caused by this figure processing error.
